# The application of Lempel-Ziv and Titchener complexity analysis for equine telemetric electrocardiographic recordings

**DOI:** 10.1038/s41598-019-38935-7

**Published:** 2019-02-22

**Authors:** Vadim Alexeenko, James A. Fraser, Alexey Dolgoborodov, Mark Bowen, Christopher L.-H. Huang, Celia M. Marr, Kamalan Jeevaratnam

**Affiliations:** 1 Faculty of Health and Medical Sciences, University of Surrey, Guildford, GU2 7AL United Kingdom; 2 Physiological Laboratory, University of Cambridge, Cambridge, CB2 3DY United Kingdom; 3 Seven Bridge Genomics, Boston, MA 02142 USA; 4 Faculty of Medicine & Health Sciences, University of Nottingham, Nottingham, NG7 2UH United Kingdom; 5 Rossdales Equine Hospital and Diagnostic Centre, Exning, CB8 7NN Suffolk, United Kingdom; 6 Division of Cardiovascular Biology, Department of Biochemistry, University of Cambridge, Cambridge, CB2 1QW United Kingdom

## Abstract

The analysis of equine electrocardiographic (ECG) recordings is complicated by the absence of agreed abnormality classification criteria. We explore the applicability of several complexity analysis methods for characterization of non-linear aspects of electrocardiographic recordings. We here show that complexity estimates provided by Lempel-Ziv ’76, Titchener’s T-complexity and Lempel-Ziv ’78 analysis of ECG recordings of healthy Thoroughbred horses are highly dependent on the duration of analysed ECG fragments and the heart rate. The results provide a methodological basis and a feasible reference point for the complexity analysis of equine telemetric ECG recordings that might be applied to automate detection of equine arrhythmias in equine clinical practice.

## Introduction

It has been widely recognised that cardiac arrhythmias pose a significant health risk for athletes, both human^1^ and equine^2,3^. They could not only impair performance but might go on to trigger potentially fatal cardiac episodes. Equine and human athletes exhibit parallel lifestyle exercise patterns taking the form of intense training which reaches its peak during races or other competitive performances and is followed by substantial reduction in intensity of exercise during subsequent lifetime retirement period. Both attain similar heart rates during individual training episodes apart from the wider equine than human heart rate variations (20 to 220 bpm).

Cardiac arrhythmias result from a diverse set of irregularities in impulse initiation and conduction or combination of both. In healthy subjects the electrical signal initiation is confined to well-defined cardiac regions such as sinoatrial or atrioventricular node, abnormalities in which result in well-defined electrocardiographic (ECG) changes. The resulting alterations in observed cardiac electrical activity may appear as directly identifiable changes in the ECG waveform both in horses and humans, particularly in the form of alterations in sinus rate, as the consequences of major anatomical disruptions in conducting or contractile tissue. More subtle ECG changes could also arise at the tissue level from atrial and/or ventricular myocardial diseases including channelopathies, alterations in calcium homeostasis and tissue heterogeneities^4^. Early detection of such changes might precede the first arrhythmic episode and therefore act as its predictor. The wide-spread use of computers and digital ECG recordings has facilitated the development of the computer-based methods of arrhythmia detection in human subjects^5–7^.

Although ECG-based screening programmes appropriate for the detection of risk of sudden cardiac deaths are available for human athletes^8^ such screening programmes do not exist for equine athletes. This in part reflects the lack of agreed classification criteria for abnormality, particularly in detection of premature complexes. Thus, subjective judgment of what is considered normal or abnormal affects the reliability of equine ECG-based diagnostics. To eliminate this subjectivity various objective measures have been proposed. In our previous paper we explored use of ECG restitution analysis to evaluate the function of equine cardiovascular system^9^. The restitution analysis has been shown to indicate the integrated mechanoelectrical impacts on the heart and of arrhythmia liability^10^. This method requires the recording of good quality ECG at high heart rates during the exercise, when electrical signal from the heart might be obscured by electrical activity of other muscles.

A more direct approach to quantify the anomalies in generation and propagation of electrical signal in the heart is to use signal complexity estimation techniques. These techniques are shown to be a sensitive tool to estimate the irregularity of various bioelectrical signals, including neuronal spiking and electroencephalographic (EEG) records^11–14^. The inherently chaotic nature of the heartbeat (as pointed out by Goldberger in 1991)^15^ makes complexity analysis a suitable tool to assess its stochasticity. Various complexity estimators were employed to identify arrhythmia^16^, to analyse circadian variability of heart rate variation^17,18^, for characterization of intermittent complexity variations during ventricular fibrillation^19^ as well as for prediction of the onset of atrial fibrillation episode^20^. Complexity estimation techniques have a benefit of not requiring high heart rate in analysed data.

These techniques are based on the seminal work of Kolmogorov^21^ who suggested that the disorderliness (complexity) of a string of a symbols might be described as the length of the shortest possible computer program capable of generating such string. Lempel and Ziv later demonstrated that complexity can be feasibly linked to the gradual build-up of new patterns along the given sequence^22^. Subsequently, a mathematical method was developed to apply the concept of Shannon entropy^23^ to repetitive time series, resulting in development of approximate entropy^24^ and sample entropy^25^ metrics, as well as some other variations, such as wavelet entropy^26^; see the recent paper of Dharmaprani *et al*.^27^ for a review. All these metrics are based on an analysis of repetitive patterns in the time series and have been used for analysis of physiological signals. In this study we primarily focus on analysis of metrics based on Lempel-Ziv string compression analyses, as it has been demonstrated that they have several-fold lower computational complexity compared to approximate entropy or sample entropy^28^ and also have no arbitrary tuning parameters which might affect their performance^29^.

The key idea of the initial Lempel and Ziv’s method (usually denoted as LZ 76) is to decompose the source string of symbols to a set of such patterns (‘terms’ or ‘factors’) which are sufficient to rebuild the source string by a machine performing copy and insertion operations^30^. The number of these patterns obtained in the decomposition step (LZ vocabulary size) is directly proportional to the complexity of the source string, and therefore to the entropy of its source. Detailed analysis of LZ decomposition method and its relation to Kolmogorov’s complexity can be found in the published literature^14,22,30–32^.

To obtain meaningful estimates of complexity values using these algorithms requires simultaneously that a sufficient amount of data is analysed and that the analysed parameter should be stationary during the data collection period^33,34^. This may lead to severe practical limitations in the time resolution of analysis in a quasiperiodic signal like the ECG as the absolute minimal amount of data is estimated to be in the range from 10 heartbeats for LZ 76 up to 80 heartbeats for sample entropy^35^. Recording of 80 equine heartbeats at rest might require up to four minutes of recording time raising the possibility of heart rate alterations originating outside of the cardiovascular system^36^.

A slightly modified and faster version of Lempel-Ziv decomposition was subsequently proposed to develop the Lempel-Ziv 1978 data compression algorithm^37^. This decomposition method (LZ 78) might be used as a complexity estimation tool as well. Both methods feature incomplete parsing of the final part of the analysed symbolic string, which introduces additional variability in the case of string shorter than 10000 symbols^33,38^. Although the error caused by this variability could be decreased by analysing longer data strings, such increase is often undesirable for analysis of bioelectrical signals as it limits its temporal resolution. An alternative way to decrease this error is to use a decomposition algorithm capable of parsing input data without any incomplete parts being left, for example one suggested by Titchener^39^.

It should be noted that although complexity estimators do produce estimates of the regularity of a signal, there is no guarantee that a particular estimator will reliably process an arbitrary type of data input. Apart from above-mentioned difficulties with analysis of “short” data sets (typical for the ECG data obtained in primary care settings), certain classes of “long” (≫10^5^ samples) data series^40^ may also be incorrectly classified by these estimators. Such problems warrant in-depth study of the suitability of a given complexity estimator to process the data before the method can be used to investigate a relevant biological problem. Probably the most important factor to be addressed in such a study would be the behaviour of an estimator in range of signal durations easily collectable in clinical practice (less than 60 seconds) and the dependence of complexity values on the heart rate.

As complexity estimation algorithms deal with sequences of symbols (strings) rather than floating-point data obtained in physiological recordings, several preparatory steps are required before the ECG can be subjected to complexity analysis. These steps typically include low-pass or band-pass filtering, baseline wander correction and resampling followed by data granulation involving conversion of the sequence of numerical ECG voltage readings to a symbolic string. Careful selection of data conditioning parameters, including sampling rate and sample length are required to ensure reproducibility of the results. The present paper describes use and feasibility of complexity estimation methods for analysis of equine ECG. In doing so we emerged with a clear methodological approach and convenient option to use in development of risk prediction algorithms for equine cardiac rhythm abnormalities.

## Results

### Dependence of ECG complexity values on the ECG strip length

**Figure 1:**
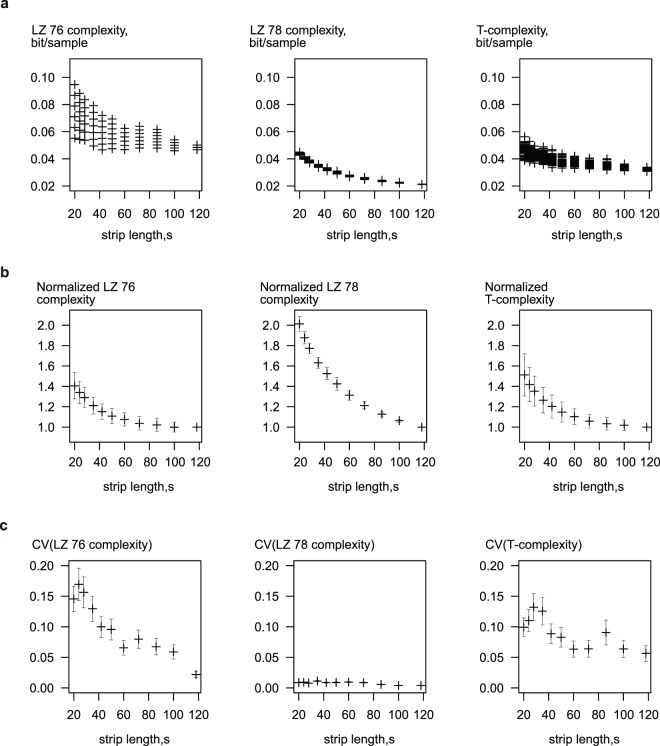
Effect of signal length on the complexity values returned by different estimators. (**a**) Distribution of complexity values obtained by analysis of different length samples extracted from a 120-sec fragment of telemetric equine ECG recording. Sixty random samples from each of the 15 horses analysed for each strip length. (**b**) Dependence of complexity values normalized to complexity at the maximal strip length (n = 15). (**c**) Effect of sample length on the coefficient of variation of different complexity values (n = 15).

To estimate the dependence of the computed complexity on the length of the analysed strip we selected a convenient sample of contiguous 120-sec ECG recordings excised from longer raw electrocardiograms of 15 individual horses. Recordings were selected to cover the range of heart rates from 32 to 145 bpm. Each 120-sec recording was then used to generate 11 sets of 60 random strips of pre-defined shorter length (20, 24, 28, 35, 42, 50, 60, 72, 86, 100 and 118 sec; total 660 strips per horse/900 strips for each strip length) which were subjected to complexity analysis. As demonstrated by a representative sample (Fig. 1a) we observed that resulting complexity values decreased in absolute value and variability with increase of the analysed strip length. To quantify the error in determination of these values we calculated ratios of complexity values obtained for the shorter strips in relation to complexity of the longest (118-sec) strips, thus eliminating dependence on the baseline complexity variations in individual subjects. The maximum increase in the shortest (20 sec) strips was up to 1.5-2-fold compared to the longest (118 sec) strips, indicating that the analysis of very short ECG strips is prone to systematic overestimation of its complexity (Fig. 1b). Additionally we have found that variation coefficients (ratio of the standard deviation to the mean which shows the extent of variability in relation to the mean of the sample) in each 60-strip group were also markedly increased in strips shorter than 60 sec (Fig. 1c). Therefore 60-second strips were used for subsequent analyses.

### Dependence of ECG complexity on heart rate

**Figure 2:**
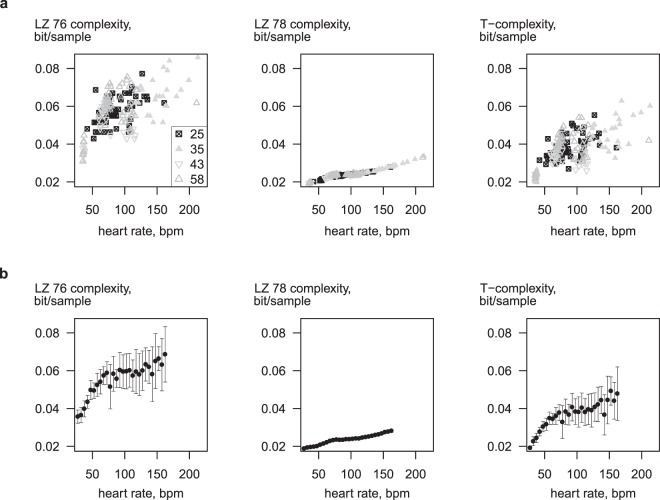
Dependence of ECG complexity on heart rate. (**a**) Representative data from four horses. (**b**) Average data from 51 horses grouped at 5 bpm heart rate ranges; only groups with three or more observations are shown.

The initial analysis of complexity dependence on the analysed strip length allowed us to evaluate the possible influence of heart rate on the ECG complexity. Due to the limitations of the data collection process for this study each subject was offering data only for a limited range of heart rates (Fig. 2a). Therefore, all available 60-sec strips extracted from all recordings were combined in one data set and grouped at 5 bpm heart rate intervals. The average LZ 76 and Titchener complexity values (but not LZ 78 values) have demonstrated the asymptotically increasing behaviour at high heart rates (Fig. 2b).

## Discussion

Our study has demonstrated that random short (60–100 sec) samples extracted from ambulatory equine ECG recordings may be used to evaluate ECG complexity. There is a clear relationship between length of the analysed strip and its complexity as well as between heart rate and complexity. All three complexity estimation methods show relatively small variabilities in their results at heart rates less than 70 bpm. At higher heart rates they exhibit a markedly different behaviour: LZ 76 complexity and T-complexity values exhibit increased spreads of complexity values with average complexity values reaching plateau. On the other hand, LZ 78 complexity values show remarkably little variation and very little increase at high heart rates. We might speculate that such different behaviour might be due to the different algorithms being sensitive to different physiological phenomena, possibly providing the foundation for the new biomarkers. The asymptotic behaviour of LZ 76 and T-complexity highlights the possibility of introduction of heart-rate corrected complexity metrics, which might be very useful to compare the data obtained at different heart rates. However, the additional theoretical and experimental studies which are required to introduce such correction formula were beyond the scope of this study. Such corrections might facilitate the direct comparison of complexity obtained at different heart rates and in different studies. The validity of such correction remains to be demonstrated in a future studies involving subjects with known pathologies associated with alterations in ECG complexity, primarily atrial fibrillation^17,41^.

Our results broadly agree with the recent study published by Zhang *et al*.^42^ in that ECG strips longer than 40 seconds are required to perform complexity analysis. In our study somewhat longer (60 sec) strips were required to stabilise complexity values. This might be due to the different coarse-graining technique we employ (beat detection instead of threshold crossing). Our choice of “beat detection” coarse-graining was primarily due to the high variability of the equine ECG and its absence of the horizontal T-P intervals which is typical for the human ECG and provides a convenient place to detect increased noise from atria. It could be expected that the high variability of the equine ECG might require development of appropriate techniques for baseline subtraction to facilitate threshold-crossing coarse-graining. Alternatively, other coarse-graining techniques might be developed for the same purpose: for example, one that relies on detection of P, Q and T wave boundaries. Such metrics would be less sensitive to noise than threshold-crossing methods and may provide more information on the excitation wave travel than beat-detection technique. Additional valuable information might possibly be supplied by emerging complexity analyses based on the second-moment statistics^43^. We expect that such easily automated analyses, probably in conjunction with currently used biomarkers might potentially be able to serve as sensitive tools for cardiovascular diagnostics. They would supplement, if not potentially replace the resource-consuming “fishing for irregular heartbeat”^44^ which is currently used for diagnosis of paroxysmal atrial fibrillation and other hard-to-detect intermittent arrhythmias in horses.

## Conclusion

The present *in vivo* study provides a methodological basis for the complexity analysis of equine telemetric ECG recordings.

## Methods

### Subject recruitment

Based on the ethical assessment review checklist by the Non-ASPA Sub-Committee at the University of Surrey, the study did not require an ethical review and received appropriate faculty level approval. Non-invasive ECG recordings were collected at Rossdales Equine Hospital and Diagnostic Centre (Newmarket, Suffolk, United Kingdom) during routine clinical work up from 51 Thoroughbred horses in race training. Data were anonymised prior to analysis. ECGs had been recorded while horses were exercised as part of their clinical investigations. All horses were of racing age at the time of testing. None showed clinically significant cardiac abnormalities.

### Data recording

Each horse was atraumatically fitted with a telemetric ECG recorder (Televet 100, Engel Engineering Services GmbH, Germany). Data were recorded in continuous episodes lasting 10–50 minutes, before and during a period of acceleration from walk to canter. This emulated incremental pacing protocol has previously been applied in studies of cardiac function *in vitro*^45,46^ and yielded ECG strips offering range of relatively steady incremental heart rates. The Televet 100 recorder has signal bandwidth of 0.05–100 Hz and sampling rate of 500 Hz.

### Data preparation

**Figure 3:**
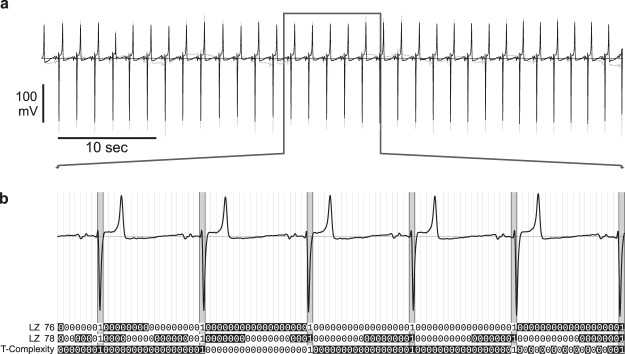
Equine ECG complexity analysis method. (**a**) Typical segment of equine ambulatory ECG. Grey, original signal, black – signal after resampling and filtration. (**b**) Conversion of ECG recording to a beat- detection binary string and subsequent complexity analyses. Beat detections shown by vertical grey bars. Decomposition of the resulting beat-detection binary string to the individual factors is indicated by alternating black/white text background. See text for the description of the decomposition methods.

The original data files were exported to CSV files with the TeleVet software. A custom software package written in GNU R, version 3.2.3^47^ was used to split the long ECG recordings and extract artefact-free 60-sec strips of lead I ECG. These strips were consequently downsampled to 125 Hz and filtered using the digital band-pass Butterworth filter. The empirically selected cut-off frequencies of 0.13 Hz and 23 Hz allowed elimination of high-frequency noise and baseline drift (Fig. 3a). The filtered recordings were converted to binary strings using a custom beat detection algorithm implemented in R (Fig. 3b). The results of the beat-detection analysis were checked for the detection errors and strips with false detections or missed beats were excluded from the subsequent analysis.

### Complexity analysis

Estimation of the binary string complexities was facilitated by a custom implementation of a complexity estimator developed in C++ for the Linux operating system, using a Qt framework (http://www.qt.io). The program simultaneously performed complexity analysis using three previously published methods: Lempel-Ziv ’76^22^, Lempel-Ziv ’78^37^ and Titchener T-complexity^39^. All three methods estimate the complexity of the symbolic string by identifying the number of sub-strings needed to build the source string (indicated by alternating black and white background in Fig. 3b). These methods differ by the algorithms of decomposition of the source strings to sub-strings; detailed descriptions of these algorithms can be found in corresponding publications. To eliminate the dependency of complexity values on the length of the source string (n), LZ 76 complexity values were normalised to the n/log_2_(n) value^30^ and LZ 78 values were normalised to sequence length. For the T-complexity, average entropy values were used.

### Statistical analyses

Parametric data are expressed as mean ± standard deviation of mean. Statistical analyses and plotting were done using GNU R^47^.
